# Lignin Based Activated Carbon Using H_3_PO_4_ Activation

**DOI:** 10.3390/polym12122829

**Published:** 2020-11-28

**Authors:** Zhongzhi Yang, Roland Gleisner, Doreen H. Mann, Junming Xu, Jianchun Jiang, J.Y. Zhu

**Affiliations:** 1 Institute of Chemical Industry of Forest Products, Chinese Academy of Forestry, National Engineering Lab. for Biomass Chemical Utilization, Key and Open Lab. on Forest Chemical Engineering, SFA, Key Lab. of Biomass Energy and Material, Nanjing 210042, China; yang166@163.com (Z.Y.); xujunming@icifp.cn (J.X.); jiangjc@icifp.cn (J.J.); 2 USDA Forest Service, Forest Products Lab., Madison, WI 53726, USA; roland.gleisner@usda.gov (R.G.); doreen.h.mann@usda.gov (D.H.M.)

**Keywords:** lignin, activated carbon, phosphoric acid activation, dye adsorption

## Abstract

Activated carbon (AC) with a very high surface area of over 2000 m^2^/g was produced from low sulfur acid hydrotropic lignin (AHL) from poplar wood using H_3_PO_4_ at a moderate temperature of 450 °C (AHL-AC6). ACs with similar surface areas were also obtained under the same activation condition from commercial hardwood alkali lignin and lignosulfonate. Initial evaluation of AC performance was carried out using nitrogen adsorption-desorption and dye adsorption. AHL-AC6 exhibited the best specific surface area and dye adsorption performance. Furthermore, the adsorption results of congo red (CR) and methylene blue (MB) showed AHL-AC6 had greater adsorption capacity than those reported in literature. The dye adsorption data fit to the Langmuir model well. The fitting parameter suggests the adsorption is nearly strong and near irreversible, especially for MB. The present study for the first time provided a procedure for producing AC from lignin with Brunauer–Emmett–Teller (BET) surface area >2000 m^2^/g using low cost and low environmental impact H_3_PO_4_ at moderate temperatures.

## 1. Introduction

Lignin is the second most abundant terrestrial biopolymer on earth, just after cellulose [1]. As a dominant aromatic polymer in nature, it accounts for approximately 15%–40% of plant biomass [2]. Isolating lignin from lignocellulosic materials in its natural unaltered state to facilitate valorization for producing biochemicals and biofuels through further processing remain a great challenge currently [3,4]. Lignin, of both native and modified forms such as commercial technical lignin, has been used as an absorbent for the removal of dyes, metal ions [5,6], and toxic compounds [7] in waste streams for clean-up. However, its adsorption capability is poor when lignin is directly used without chemical modifications [8]. Through carbonization and activation to prepare highly porous activated carbon (AC), lignin-based materials exhibits superior absorption performance [9].

AC is the oldest adsorbent known, having a very porous structure with a large internal surface area and possessing good adsorption capacities towards various substances. Industrial demands for AC are high and growing, and so does the production of AC. Approximately 80%–85% of total AC production is derived from non-renewable coal-based resources with the remaining from renewable sources such as agricultural residues, wood, and coconut shells. As a hydrocarbon macromolecule with a carbon content of greater than 60%, lignin can meet the high carbon yield requirement for commercial AC production. Furthermore, lignin tends to form graphite-like domains during activation due to its network structure of aromatic ring resulting in AC suitable for electrodes in batteries, with good electrochemical performance [10]. Therefore, lignin can be an ideal precursor for producing high grade AC for high value applications in addition to low grade absorbents [8]. In comparison with coal-derived ACs, lignin-based ACs can reduce the carbon footprint because commercial technical lignin is derived from wood through pulping.

There are two different processes for preparing ACs: Physical and chemical activations. Nevertheless, chemical activation has shown more advantages for producing ACs with higher yield and higher surface area at moderate temperatures using only one step compared with physical activation [11]. The most commonly used chemical activation agents are H_3_PO_4_, alkali metal compounds (NaOH, KOH, Na_2_CO_3_, and K_2_CO_3_), and ZnCl_2_ [12]. Alkali such as KOH is often carried out at high temperatures of 700–900 °C with an impregnation ratio (IR = carbon biomass:catalyst) = 1:2–5 [13,14] and is very effective in creating micropores, small mesopores, and, therefore, very high specific Brunauer–Emmett–Teller (BET) surface area of above 2000 m^2^/g. However, KOH activation has low carbon yield and has detrimental impact to human health and environment [15]. H_3_PO_4_ on the other hand, can be utilized at low temperatures 400–600°C with a low IR of 1:1–3, resulting in BET surface area of approximately 1000–1500 m^2^/g consisting of mainly mesopores [16,17,18]. H_3_PO_4_ as a catalyst not only promotes bond cleavage reactions but also facilitates crosslinking via cyclization, condensation, and forming a layer of linkage such as phosphate and polyphosphate esters which could protect the internal pore structure and thus prevent excessive burn-off in carbon activation [19]. With the considerations of environmental impact, energy cost, and carbon yield, H_3_PO_4_ is highly attractive and has been increasingly used in large-scale AC production in recent decades [20].

Generally speaking, activated carbons with BET surface area below 2000 m^2^/g are widely used for applications associated with low-cost absorption such as heavy metals removal, dyes adsorption, and air pollution control. ACs with specific BET surface area greater than 2000 m^2^/g are considered as super activated carbon [21] with higher added value for applications such as catalyst carrier, gas (CH_4_, H_2_, and CO_2_) storage materials, electrode for supercapacitors, and adsorbents requiring high adsorption capacity [22]. The present study evaluates the potential of using low cost H_3_PO_4_ activation for producing super activated carbon from different lignin samples including several commercial lignin samples. The novelty of the present study are two-fold: (1) focusing on producing AC with specific BET surface area of greater than 2000 m^2^/g for high value applications using low cost H_3_PO_4_ activation; and (2) examining lignin from low temperature acid hydrotropic fractionation (AHF) [23] that is metal-free and theoretically sulfur-free for producing super AC. AHF was a recently developed fractionation process and has the advantage of rapid dissolving lignin and hemicelluloses at atmospheric pressure and low temperatures with high selectivity [23]. When using maleic acid as an acid hydrotrope in AHF [24,25], sulfur-free lignin can be obtained. Majority of the commercial technical lignin contain sulfur, metal, and ammonia cations from sulfite and sulfate pulping. The release of sulfur and ammonia during activation can have negative environmental impacts. Therefore, evaluation of sulfur-free acid hydrotropic lignin (AHL) has environmental significance.

## 2. Experimental

### 2.1. Materials

Poplar NE222 (Populus deltoides Bartr. ex Marsh. × P. nigraL.) was harvested from the Hugo Sauer Nursery of USDA Forest Service, Northern Research Station, Rhinelander, WI, USA. Wood logs were manually debarked, chipped, and then Wiley-milled to 20-mesh powder at the Forest Products Laboratory, USDA Forest Service, Madison, WI, USA. The milled poplar wood was air dried at room temperature to approximately 11% moisture content. *p*-TsOH (ACS reagent, ≥ 98.5%), H_3_PO_4_ solution (85 wt.%), alkali lignin from spruce (softwood), along with the adsorbates, congo red (CR, dye content: ~40%) and methylene blue (MB, dye content: 97%), were all purchased from Sigma-Aldrich (St. Louis, MO, USA). The molecular structure and physical properties of CR and MB are listed in Table S1. Sulfuric acid (98 wt.%) and hydrochloric acid (36.5–38 wt.%) were purchased from Fisher Chemical (Fisher Scientific, Pittsburgh, PA, USA). Indulin C, functionalized with carboxylic acid, aliphatic, and aromatic hydroxyl groups, and free of reducing sugars, was obtained from Westvaco Corp (now WestRock). A commercial lignosulfonate from hardwood was complimentary provided by LignoTech, a subsidiary of Borregaard, Rothschild, WI, USA.

### 2.2. Preparation of Theoretically Sulfur-Free AHL

**Figure 1:**
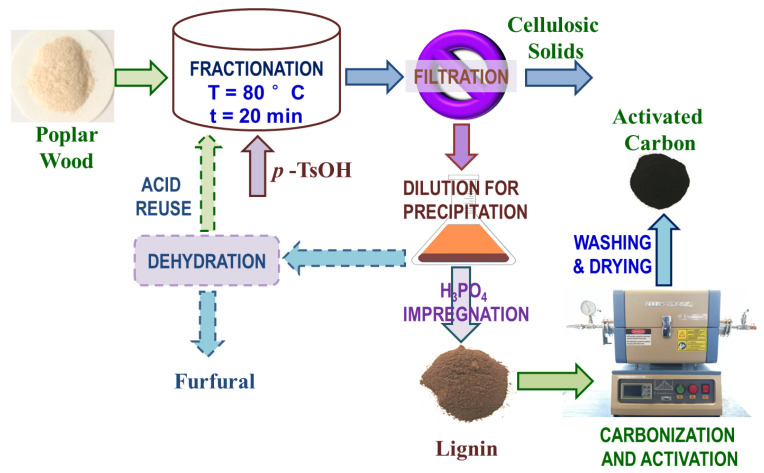
Experimental flow schematic diagram showing wood fractionation, lignin separation, and activation for producing activated carbon. Processes connected with dashed arrows were not carried out in this study.

AHF of Wiley-milled poplar wood using *p*-TsOH [23,26] was carried out according to the experimental schematic flow diagram shown in Figure 1 to produce theoretically sulfur-free AHL for activation study. The 500 g *p*-TsOH solution at 80 wt.% concentration was prepared in a 1000 mL flask by solubilizing required amounts of *p*-TsOH in deionized (DI) water. The flask was placed on a temperature-controlled shaker (Model 4450, Thermo Scientific, Waltham, MA, USA) at 250 rpm to promote *p*-TsOH dissolution. Then, 50 g (air dry basis) poplar sample was fractionated in the prepared *p*-TsOH solution with continuous shaking at 80 °C for 20 min. At the end of the preset reaction time, 1 L DI water was added into the flask to dilute the fractionation solution to terminate reaction. The water insoluble solids (WIS) from fractionation were separated from the AHF spent liquor through filtration. The filtrate was diluted to 5 wt.% *p*-TsOH concentration to precipitate lignin. After centrifuging, the precipitated lignin was dialyzed in DI water for one week and oven dried at 50 °C for later activation. The lignin-free spent liquor can be used for producing furfural and recycling *p*-TsOH [26].

### 2.3. AC Preparation

AHL and other commercial technical lignin were activated for producing ACs. Lignin (2 g) was impregnated in H_3_PO_4_ solution (85 wt.%) at the varied IR (Lignin: H_3_PO_4_) along with 0.3 g sulfuric acid solution (72 wt.%) in a quartz boat. After evenly stirring, the mixture was put into an oven at 85 °C for 12 h. The mixture was then placed in a tube furnace under the self-generated atmosphere (open to atmosphere). The furnace was heated to 200 °C at 5 °C/min, and held for 60 min. It was then heated to a final activation temperature at 2.5 °C/min and held for a preset period to activate lignin before cooling down to 200 °C at 5 °C/min followed by natural cooling to room temperature. The activated sample was washed with a small amount hydrochloric acid and hot water until neutral, and then dried at 105 °C for 4 h. After cooling down to room temperature in a desiccator, the sample was weighed to determine yield. Finally, the AC sample was ground into powder in a crucible.

### 2.4. Characterizations

The surface functional groups of AHL and ACs were analyzed using a Fourier transform infrared (FTIR) spectrometer (Spectrum Two, PerkinElmer, Waltham, MA, USA) equipped with a universal attenuated-total-reflection (ATR) probe.

The specific surface area and pore distribution of ACs were determined by N_2_ adsorption/desorption at 77 K, after degassing at 120 °C for 6 h, using a surface area analyzer (TriStar II, Micromeritics Instrument Corp., Norcross, GA, USA). Briefly, the surface area was calculated by the BET model from relative pressures (P/Po) in the range of 0.01–0.35; the total pore volume was obtained by single-point adsorption of N_2_ at a high relative pressure (0.99); micropore area and micropore volume were determined by the t-plot method; mesoporous area and mesoporous volume were calculated using the Barrett–Joiner–Halenda (BJH) model; pore size distribution was auto-generated by applying density functional theory (DFT) to the N_2_ adsorption isotherms using the software supplied by the TriStar II. According to the IUPAC classification, the pore diameter of micropores are less than 2 nm, mesopores 2–50 nm, and macropores > 50 nm. Replicate runs were conducted for a few samples and excellent repeatability was obtained (Table S2)

The surface functional groups and atomic elements were detected by X-ray photoelectron spectroscopy (XPS) on a spectrophotometer (K-Alpha+, ThermoFisher Scientific, Waltham, MA, USA) with a monochromated Al Ka X-ray source and used C1s (284.8 eV) as standard to correct for the other peaks.

### 2.5. Adsorption Experimentation

CR and MB solutions in a range of concentrations were prepared using DI water. The adsorption behaviors of ACs were investigated according to the following procedure. A 50 mL CR or MB solution of a known concentration was poured into a flask, and then a specified amount of AC (5, 10, 15, 20, and 25 mg) was added into the solution. All bottles were placed into a temperature-controlled shaker (Excella E25, New Brunswick Scientific, Edison, NJ, USA) at 150 rpm and ambient temperature for 48 h to obtained adsorption equilibrium. The resultant supernatant of the solution was subsequently extracted through centrifugation at 10,000 rpm for 5 min, and the absorbance of the supernatant was measured by a UV-vis spectrophotometer (model 8453, Agilent Technologies, Palo Alto, CA, USA) at the wavelength listed in Table S1. Both CR and MB dye solutions with known concentrations were used to develop calibration equations (Equations (S1) and (S2)).

## 3. Results and Discussion

### 3.1. N_2_ Adsorption-Desorption of AC Isotherms

**Figure 2:**
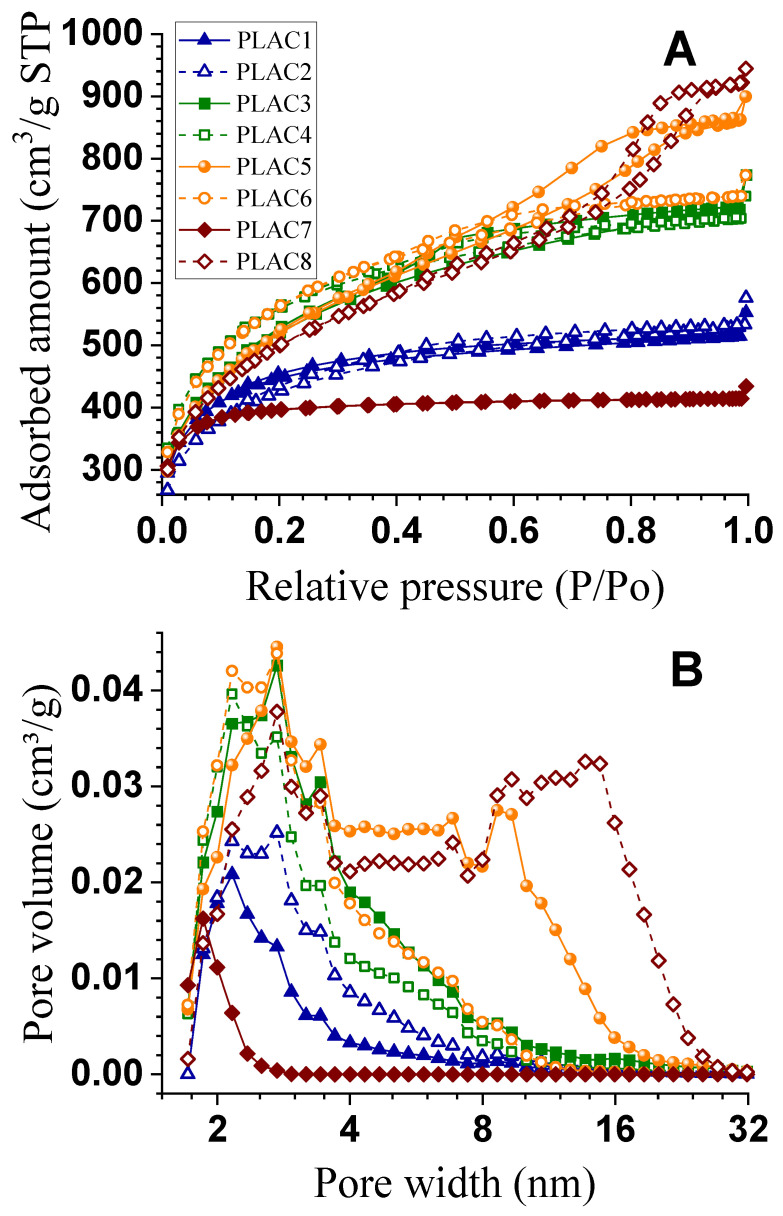
(**A**): Nitrogen adsorption-desorption isotherms of 8 activated carbons (ACs); (**B**): Pore size distributions of 8 ACs.

N_2_ adsorption-desorption studies of AC samples from poplar AHL prepared under different activation temperatures, times, and impregnation ratios using H_3_PO_4,_ as listed in Table 1, were carried out and the results are shown in Figure 2A. According to the IUPAC classification, the N_2_ adsorption-desorption isotherms of AHL-AC7 belonged to type I, indicating AHL-AC7 primarily contains micropores. The isotherms of AHL-AC5 and AHL-AC8 belonged to type IV with hysteresis loops, indicating the two ACs contain primary mesopores. The isotherms of AHL-AC1, -AC2, -AC3, -AC4, and -AC6 are a mixture of type I and type IV with hysteresis loops, indicating the co-existence of micropores and mesopores in these ACs. In the low-pressure region where P/Po ≤ 0.1, N_2_ uptake was obvious with rapid increase in adsorption capacity with increasing pressure, an adsorption characteristic of materials with micropores. In the high-pressure region, hysteresis loops were present due to the capillary condensation of adsorbate in slit-shaped mesopores in ACs [27]. AHL-AC5 and -AC8 exhibit the highest adsorption capacity among all the samples examined. AHL-AC6 has excellent adsorption capacity in the low and middle pressure regions, suggesting the existence of well-developed micropores and small mesopores. AHL-AC5 and -AC8, however, have greater absorption capacity in the high-pressure region than other ACs, indicating the presence of considerable larger mesopores, and the multi-molecular layer adsorption in the larger mesopores.

The AC pore size distributions calculated by DFT were shown in Figure 2B. AHL-AC1, -AC2, -AC3, -AC4, -AC6, and -AC7 contained mainly pores in the range of 2–8 nm and which accounted for most of the pore volumes, indicating these ACs consist of pores in the region of small mesopores to near micropores. AHL-AC3 and -AC6 have the greatest incremental volumes among these samples. AHL-AC5 and -AC8 are more mesoporous with pore volume distributions of AHL-AC5 ranging from 2–16 nm while AHL-AC8 is in the range of 2–24 nm. AHL-AC5 and -AC8 are advantageous for adsorbing large molecules.

AC yields were calculated based on the amounts of starting AHL as listed in Table 1 along with measured BET surface area (S_BET_). AHL-AC7 has the smallest S_BET_ = 1321 m²/g, perhaps due to short activation time of 1 h and low H_3_PO_4_ loading in impregnation with IR = 1:1. AHL-AC6 has the highest S_BET_ = 2015 m²/g. It appears that an activation temperature around 450 °C for approximately 1.5 h with IR about 1:2 produced ACs (AHL-AC3, -AC5, -AC8) with excellent BET surface area. AHL-AC8 has the highest mesopore volume 1.13 cm^3^/g and the largest average pore diameter 4.33 nm. In general, all of the prepared AHL-ACs using H_3_PO_4_ activation, excepting AH-AC1 and -AC7, contain mainly mesopores, although both micro and mesopores, are present. In contrast, AH-AC1 and -AC7contain a larger percentage of micropores than mesopores. AHL itself is almost nonporous, with a very low S_BET_ = 0.24 m^2^/g.

We compared AHL-AC6 with ACs from three commercial technical lignins produced under the same condition as AHL-AC6 (Table 1). IndL-AC, prepared from Indulin C with a very high O content (Table S3), had a lower yield of 45%, lower S_BET_ = 1371 m^2^/g, and a smaller total pore volume of 0.66 cm^3^/g. The AlkL-AC from alkali lignin (softwood) has similar properties as AHL-AC6 with almost the same yield. The LS-AC from a commercial lignosulfonate has a slightly higher S_BET_ = 2179 m^2^/g than that of AHL-CA6 = 2015 m^2^/g, and a significantly greater total pore volume of 1.81 cm^3^/g and mesopore volume of 1.65 cm^3^/g than the corresponding values for AHL-AC6. However, LS-AC only had a 32% yield compared to the 62% for AHL-AC6, perhaps due to the higher sulfite (${\mathrm{SO}}_{3}^{-2}$) of approximately 13%, and carbohydrate content in the LS as can be seen from the higher S and O content in surface elemental composition analysis (Table S3).

We also compared the ACs produced in this study with those reported in literature using lignin as a precursor. As can be seen in Table 2, all the cited work using H_3_PO_4_ for activation reported substantially lower S_BET_ < 1500 m^2^/g and total pore volume V_tot_ < 1.0 cm^3^/g, in comparison with S_BET_ = 2015 m^2^/g and V_tot_ = 1.2 cm^3^/g for AHL-AC6 and S_BET_ = 2119 m^2^/g and V_tot_ = 1.1 cm^3^/g for AlkL-AC, despite using either higher temperatures, higher dosages of H_3_PO_4_, or inert gases. The best BET surface area achieved was 1459 m^2^/g [33] using a moderate temperature of 425 °C and activation time similar to those used in the present study, but using a higher heating rate and under N_2_ treatment rather than under self-generated atmosphere. Alkali activation is known to create micropores, small mesopores with high specific BET surface area of 3235 m^2^/g [37]. However, its low carbon yields and impacts on environment make it less attractive in the future.

Many factors affect AC properties. In H_3_PO_4_ activation, reactions such as condensation, deoxygenation, dehydration, and cross-linking can occur depending on two processes: Impregnation and heat treatment. Impregnation temperature is an important factor on the interaction of H_3_PO_4_ and lignin macromolecules, which directly affects activation. Significant alteration of lignin structure was observed using impregnation at 50 °C [38]. ACs with high S_BET_ and large volume were obtained using impregnation temperatures of 80 °C and 85 °C, respectively [39,40]. Guo et al. found that adding a small amount of concentrated H_2_SO_4_ solution could promote the penetration of H_3_PO_4_ into raw materials and increase the S_BET_ and pore volume of AC [41,42]. Therefore, we chose 85 °C and supplemented a small amount of sulfuric acid into the H_3_PO_4_ solution for impregnation. Heat treatment is determined by activation atmosphere, the heating rate, the intermediate and final temperatures, and the corresponding residence times. Self-generated atmosphere was found to produce ACs with a greater S_BET_ and yield than N_2_ and air flow from coconut shell under H_3_PO_4_ activation [43]. Furthermore, oxygen derived from the gas phase may play an important positive role in activation as coking of volatiles can contribute to the formation of the porous structure. We therefore chose self-generated atmosphere in the present study. Furthermore, the intermediate treatment temperature 200 °C in the first phase of the two-step heat treatment slows the heating rate and favors pore development through the formation of graphite-like structures. The moderate temperature of 450–500 °C in the second step facilitates maximal pore development [44,45] by decreasing phosphate groups and simultaneously increasing graphite-like structures on the surface of carbonized materials [46].

### 3.2. FT-IR Analysis

**Figure 3:**
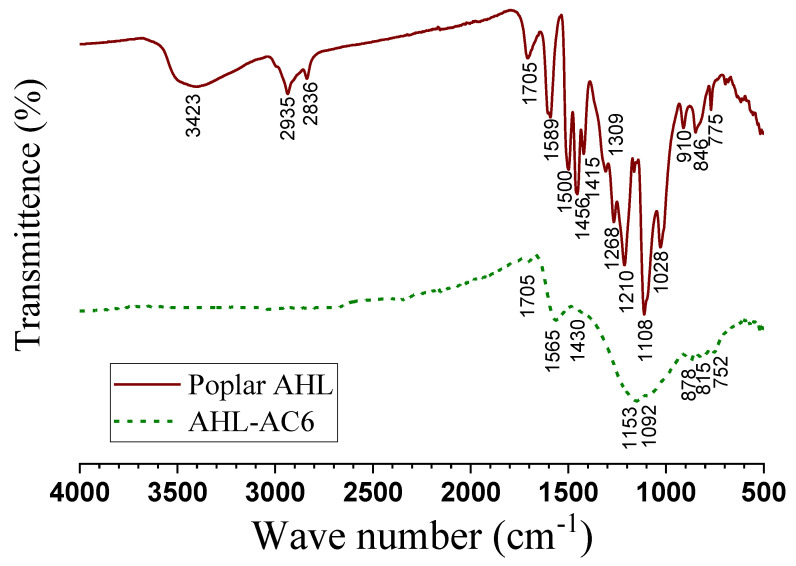
FT-IR spectra of acid hydrotropic lignin (AHL) and AHL-AC6.

The FT-IR analyses show that AHL-AC6 has less absorption bands than does AHL, indicating that some functional groups present in the poplar AHL were lost through carbonization. As shown in Figure 3, the aliphatic C–H stretching bands at 2935 and 2836 cm^−1^ and the C–H bending vibrations of methylene and methyl at 1456 and 1309 cm^−1^ in the AHL spectrum were not detected in the spectrum of AHL-AC6, indicating a considerable decrease in aliphaticity after carbonization [47]. The strong bands of aromatic ring stretching at 1589 cm^−1^ and 1500 cm^−1^ enhanced by polar functional groups, such as –OH, shown in the AHL spectrum were shifted to 1565 cm^−1^ with reduced intensity in the AHL-AC6 spectrum. This frequency shift can be explained by aromatic ring condensation. The decrease in peak intensity may result from the elimination of –OH groups, consistent with the disappearance of the peak at 3423 cm^−1^ in AHL-AC6 spectrum. The band at 1705 cm^−1^ is attributed to carbonyl groups. More carbonyl groups are formed as a result of chemical activation. The formation of carbonyl groups in AHL-AC6 corresponds to the disappearance of aromatic C–O stretching at 1268 and 1210 cm^−1^ in the AHL spectrum. This suggests that the formation of carbonyl groups is from the cleavage of oxygen-containing linkages in AHL.

Bands at 1153 and 1092 cm^−1^ in the spectrum of AHL-AC6, but not in the AHL spectrum, are characteristics of phosphorous and phosphor carbonaceous compounds present in AHL-AC6. The 1153 cm^−1^ band corresponds to the stretching of hydrogen-bonded P=O and C–O stretching vibration in C–O–P, while the 1092 cm^−1^ band is related to P–O–P stretching [48,49].

### 3.3. XPS Analysis

**Figure 4:**
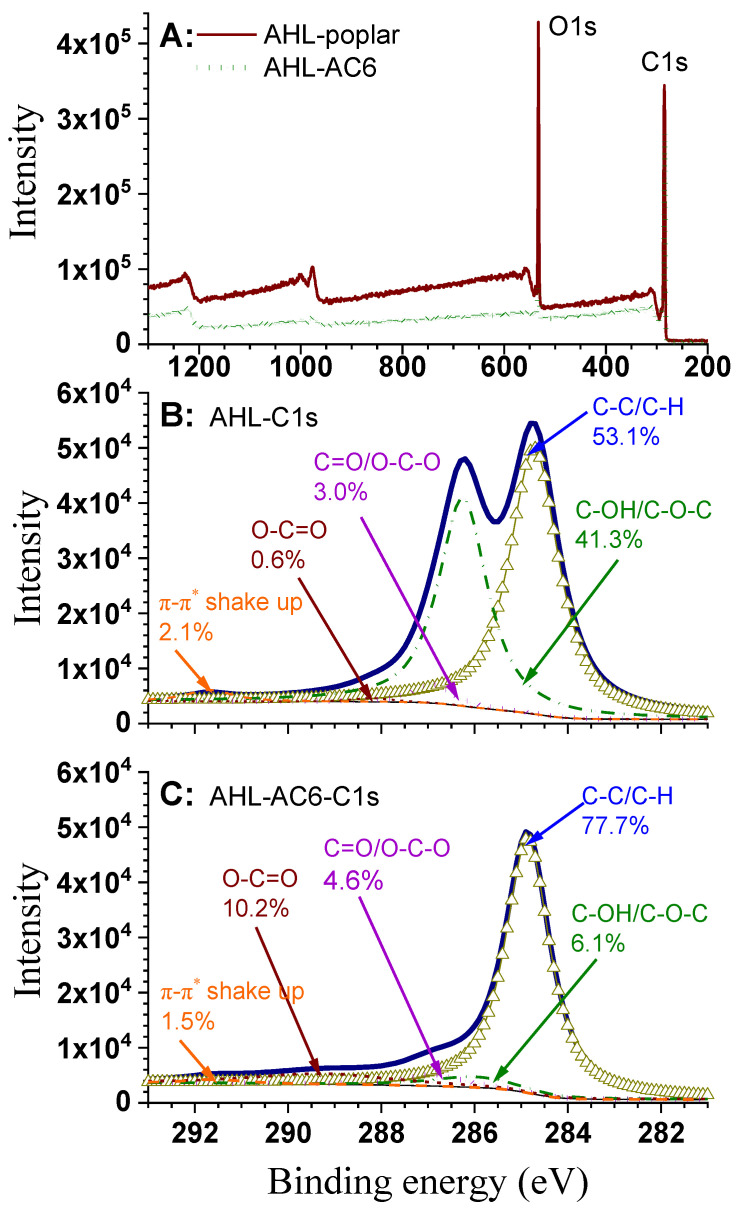
XPS spectra of AHL and AHL-AC6 (**A**); C1s region for AHL (**B**), and C1s region for AHL-AC6 (**C**).

The XPS spectra of poplar AHL and AHL-AC6 show two major peaks ascribed to C1s and O1s photoelectrons (Figure 4A). S in AHL-AC6 was enriched due to the loss of elemental oxygen through carbonization. The surface elemental composition based on XPS measurements (Table 3) indicate AHL has only 0.22% S from the residual *p*-TsOH in the sample and H_2_SO_4_ used for impregnation. The presence of P is from the H_3_PO_4_ used for impregnation. Carbonization substantially reduced elemental oxygen from 25.1% to 8.5% to result in enriched carbon content of 90.4% from 70.6%.

In order to determine the functional groups related to C on samples surface, the AHL and AHL-AC6 C1 peaks in high resolution were deconvoluted as shown in Figure 4B and C, respectively. The C–C/C–H of graphitic carbon (C1, 284.5 to 285.0 eV) [50,51] was the main contributing component to the C1 peak for both samples. However, the contribution of graphitic carbon was over 70% in AHL-AC6 (Figure 4C), substantially higher than the 53.4% in the unactivated lignin AHL (Figure 4B). This can be attributed to [52]: (1) heat treatment of lignin leads to homolytic cleavage of the alkyl-aryl ether linkages such as β–O–4′ to form a direct linkage between the aromatic ring and the neighboring aliphatic chain, and (2) the conversion of C2 into C1 by releasing CO_2_. C–OH/C–O–C representing phenolic, alcohol, ether, or C=N groups (C2, 285.5 to 286.2 eV) [50,53] was substantially reduced through activation, i.e., from 34.0% (Figure 4B) in AHL to only 9.2% (Figure 4C) due to oxidation. The change in the relative content of C=O/O–C–O in carbonyls and quinones (C3, 287.2–287.9 eV) [50,53] was insignificant. Activation also increased O–C=O associated with carboxyl or ester groups (C4, 288.1 to 289.1 eV) [50,53]. Direct oxidation can transform the phenolic-lignin end-groups and terminal hydroxymethyl groups into carboxyl groups [52]. The peak at 291.2 eV was attributed to shake-up satellite due to π–π* transitions in aromatic rings (291.1 to 291.6 eV) [50]. The intensity of this peak was related to the graphitization degree of materials [54]. The change in the relative content indicates the increasing concentration of condensed aromatics on the surface of AHL after carbonization [55].

### 3.4. Dyes Adsorption

**Figure 5:**
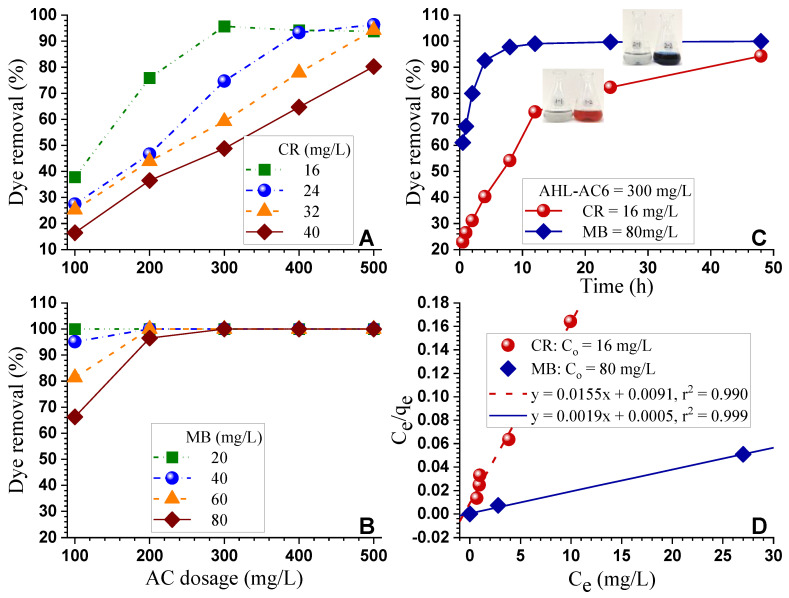
Dye adsorption by AHL-AC6. Dye congo red (CR) (**A**) and methylene blue (MB) (**B**) removal capacity after 48 h adsorption. At AC dosage of 300 mg/L dye solution, dye removal with respect to absorption duration (**C**) and adsorption isothermal fit to Langmuir model (**D**).

The adsorption of CR and MB by AHL-AC6 was evaluated in a range of dye concentrations and AC loadings. The results (Figure 5) indicate that the percentage of dye removal is linearly proportional to AC dosage at all dye concentrations until reaching 100% dye removal. Using AC dosage of 300 mg/L can remove 100% CR at 16 mg/L (Figure 5A). AC dosage of only 200 mg/L is sufficient to remove 100% MB ≤ 60 mg/L (Figure 5B). These results clearly indicate AHL-AC6 preferentially adsorbing MB to CR. The CR molecular size of 2.3 nm [56] is greater than the MB size of 1.4 nm [57], resulting in a lower extent of diffusion into the pores of AHL-AC6 and therefore a lower amount of adsorption.

Adsorption experiments show that initial adsorption of dye CR and MB by AHL-AC6 were rapid (Figure 5C). The initial rate of adsorbing MB was greater than adsorbing CR. This is also related to the smaller MB molecular size than that of CR as discussed above. The adsorption rate for MB decreased rapidly once it reached 90% dye removal at 4 h. Adsorption for CR with respect to adsorption duration was linear with two rates transitioning at approximately 12 h when CR removal achieved approximately 75%. The rapid initial adsorption was attributed to the numerous available adsorption sites on the AC surface. As absorption progressed, the AC became saturated with dye, which decreased the rate of adsorption. The rapid initial adsorption was attributed to the numerous available adsorption sites on the AC surface. As absorption progressed, the AC became saturated with dye, which decreased the rate of adsorption.

The dye adsorption data were easily fit to the Langmuir (Equation (S6)) model (Figure 5D), as can be seen from the *r^2^* values and the small differences between the measured and theoretical Langmuir maximal adsorption capacity *q_m_* listed in Table 4. The data fit poorly to the Freundlich model (Equation (S9), Figure S1, Table S4), different from some literature [58]. The very low *R_L_* values in the Langmuir model suggest that the adsorption is strong [59] and almost irreversible, especially for MB.

The comparisons of the calculated and experimental maximal adsorption capacities *q_m_* for dye CR and MB by AHL-AC6 were compared with literature data (Table S5). The results indicate that the adsorption capacity of AHL-AC6 for CR is relatively ordinary, but for MB, the adsorption ability is remarkable compared with literature data.

**Figure 6:**
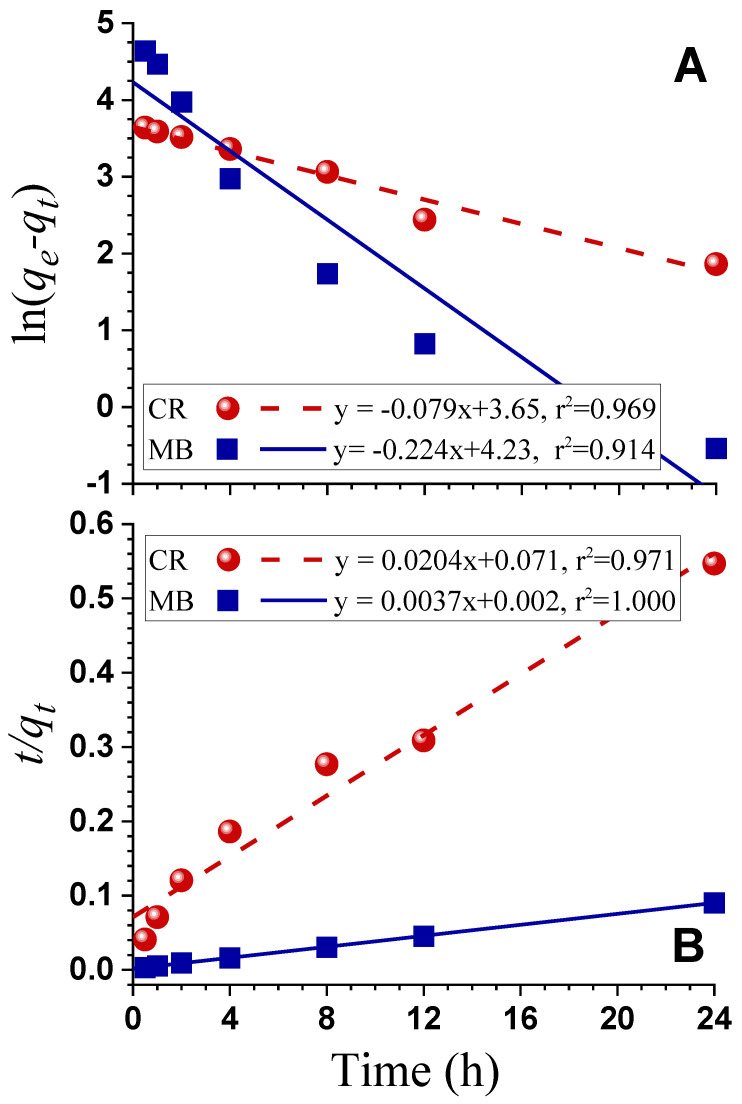
AHL-AC6 kinetic dye adsorption data fitted to models. (**A**): Pseudo-first order model; (**B**): Pseudo-second order model. Initial dye concentrations: CR C_o_ = 16 mg/L; MB C_o_ = 80 mg/L. Amount of AHL-AC applied = 300 mg/L.

The pseudo-first order kinetic model (Equation (S10)) and the pseudo-second order kinetic model (Equation (S11)) were used to investigate the adsorption mechanism of dye CR and MB by AHL-AC6. The CR adsorption fit well to both the pseudo-first order and the pseudo-second order kinetic model (Figure 6, Table S6), suggesting that the adsorption rate is not only dependent on the concentration of CR or the amount of AHL-AC6 applied, but also related to the available adsorption sites and the time to attain equilibrium [60,61]. The MB adsorption by AHL-AC6 fit well only to the pseudo-second order kinetic model, suggesting that the rate of adsorption is only dependent on the available adsorption sites and the time to attain equilibrium saturation on the AHL-AC6 surface [60].

## 4. Conclusions

A low sulfur (theoretically sulfur-free) lignin from acid hydrotropic fractionation using *p*-TsOH was used to prepare activated carbon with excellent adsorption performance. ACs with high yields of 60.1%–67.5% were obtained using low cost H_3_PO_4_ as an activation agent under moderate temperatures. A BET surface area of over 2000 m^2^/g, substantially higher than <1500 m^2^/g reported in literature, was obtained under the impregnation ratio 1:2 activated at 450 °C for 1.5 h. Similar high BET surface areas were also obtained from commercial alkali lignin and lignosulfonate. Data from congo red and methylene blue dye adsorption using the AC with greatest BET can be fit to the Langmuir model well, with maximum adsorption capacity of CR and MB of 65 and 535 mg/g, respectively. The adsorption process of CR and MB follows the pseudo-second order kinetics. The AC obtained from prepared poplar lignin has a more favorable effect on the adsorption of MB than CR. The significance of the present work lies in the fact that ACs with BET surface area greater than 2000 m^2^/g can be produced from hardwood acid hydrotropic lignin that can be made sulfur-free, as well as commercial hardwood technical lignins, using the low cost and low environmental impact activation agent H_3_PO_4_.

## Figures and Tables

**Table 1:** Microstructural properties of ACs prepared under different activation conditions.

Samples	T (°C)	t (h)	IR	Yield (%)	S_BET_ (m²/g)	V_tot_ (cm³/g)	V_micro_ (cm³/g)	V_meso_ (cm³/g)	D (nm)
AHL-AC1	350	1	1:2	67.5	1526	0.86	0.36	0.29	2.88
AHL-AC2	350	1.5	1:3	70.0	1480	0.89	0.25	0.43	2.97
AHL-AC3	400	1.5	1:3	66.2	1871	1.20	0.16	0.75	3.08
AHL-AC4	400	2	1:2	64.8	1956	1.14	0.23	0.60	2.92
AHL-AC5	450	1	1:3	64.2	1869	1.39	0.12	1.05	3.69
AHL-AC6	450	1.5	1:2	62.3	2015 ± 5	1.20	0.19	0.73	2.94
AHL-AC7	500	1	1:1	60.1	1321	0.67	0.51	0.08	2.86
AHL-AC8	500	2	1:3	64.3	1790	1.46	0.16	1.13	4.34
AHL	-	-	-	-	0.24	0.0003	-	-	4.37
IndL-AC	450	1.5	1:2	45.3	1371 ± 25	0.66	0.36	0.19	3.82
AlkL-AC	450	1.5	1:2	61.8	2119	1.13	0.19	0.66	2.61
LS-AC	450	1.5	1:2	32.3	2179	1.81	-	1.65	3.41

S_BET_: Brunauer–Emmett–Teller (BET) surface area, V_micro_: Micropore volume, V_meso_: Mesopore volume, V_tot_: Total pore volume, and D: Average pore diameter.

**Table 2:** Comparisons of lignin-based ACs from the presents study with those reported in literature.

Material	Conditions	S_BET_ (m²/g)	V_tot_ (cm³/g)	V_micro_ (cm³/g)	V_meso_ (cm³/g)	Source
Brew spent grain hydrolysis lignin	H_3_PO_4_, IR = 1:3, impregnation for 1 h at room temperature, 17.5 °C/min to 170 °C for 1 h, 600 °C for 2 h in air	459	0.30	0.17	0.13	[28]
Softwood sodium lignosulfonate	H_3_PO_4_ (60%), IR = 1:1, impregnation for 1 h at 110 °C, 10 °C/min to 1000 °C in argon	1373	0.97	0.41	0.56	[29]
Alcell^®^ lignin	H_3_PO_4_, IR = 1:3, impregnation for 24 h at 60 °C in a vacuum dryer, 500 °C for 2 h in N_2_	1015	-	0.37	1.10	[30]
Kraft lignin	H_3_PO_4_, IR = 1:1.4, impregnation for 1 h at room temperature, 10 °C/min to 150 °C for 1 h, 600 °C for 2 h in air	1305	0.67	-	-	[31]
Kraft lignin	H_3_PO_4_, IR = 1:1.5, microwave pretreatment for 4 min, 600 °C for 1 h in a closed stainless steel reactor	1202	0.66	0.48	0.16	[32]
Eucalyptus kraft lignin	H_3_PO_4_, IR = 1:2, impregnation for 24 h at 60 °C in a vacuum dryer, 10 °C/min to 425 °C for 2h in N_2_	1459	-	0.82	0.53	[33]
Eucalyptus kraft lignin	CO_2_, 10 °C/min to 350 °C for 2 h in N_2_, then 850 °C for 20 h in CO_2_	1853	2.09	0.70	0.86	[34]
Eucalyptus kraft lignin	ZnCl_2_, IR = 1:2.3, impregnation in a rotary evaporator for 1 h at 30 °C and vacuum-evaporation at 60 °C, 10 °C /min to 500 °C for 2 h in N_2_	1321	0.79	0.06	-	[35]
De-alkaline lignin	KOH, IR = 1:3, 10 °C/min to 800 °C for 5 min in N_2_	2254	1.14	1.02	-	[36]
Lignin waste	KOH, converted to hydrochar via hydrothermal treatment at 300−390 °C, IR = 1:4, 3 °C/min to 800 °C for 1 h in N_2_	3235	1.77	0.93	-	[37]
Poplar AHL	H_3_PO_4_ (aliquot H_2_SO_4_), Condition for AHL-AC4 (Table 1)	1956	1.14	0.23	0.60	This study
Poplar AHL	H_3_PO_4_ (aliquot H_2_SO_4_), Condition for AHL-AC6 (Table 1)	2015	1.20	0.19	0.73	This study
Alkali lignin	H_3_PO_4_ (aliquot H_2_SO_4_), Condition for AHL-AC6 (Table 1)	2119	1.13	0.19	0.66	This study

**Table 3:** Elemental analysis of AHL and AHL-AC6 by XPS.

Samples	C (%)	O (%)	S (%)	P (%)	O/C
AHL	74.56	25.09	0.22	0.13	0.34
AHL-AC6	90.39	8.49	0.52	0.60	0.09

**Table 4:** Parameters of the Langmuir isotherm models for CR and MB adsorption by AHL-AC6.

Dye	q_m_ (mg/g)	k	R_L_	r^2^	Experimental q_m_ (mg/g)
CR	64.7	1.7045	0.0354	0.9904	60.7
MB	534.8	3.8998	0.0032	0.9985	530.1

## Supplementary Materials

The following are available online at https://www.mdpi.com/2073-4360/12/12/2829/s1, Figure S1: Adsorption isotherms of CR and MB by AHL-AC6 fit to the Freundlich model, Table S1 Molecular structure and physical properties of the used dyes, Table S2 Repeatability test of BET surface area measurements, Table S3 XPS measurements of surface elemental composition of lignin samples, Table S4 Parameters of the Freundlich isotherm models for CR and MB adsorption by AHL-AC6, Table S5 Comparisons of dye adsorption capacity by AC among different studies, Table S6 Parameters for the pseudo-first order and the pseudo-second order model equations of CR and MB adsorption.
