# Supplementary material for: Lignin Based Activated Carbon Using H3PO4 Activation
Source: Polymers (Basel). 2020 Nov 28;12(12):2829. doi: 10.3390/polym12122829 (PMC7760334; doi:10.3390/polym12122829)
Supplement: Supplementary file 1 [file polymers-12-02829-s001.pdf]

polymers

Supplementary Information for

Preparation of high surface area activated carbon using low cost H_3PO_4 at moderate temperatures from acid hydrotropic lignin

Table S1. Molecular structure and physical properties of the used dyes.

Dye	Molecular formula	Structure	Type	Molecular weight (g/mol)	Adsorption peak (nm)
Congo red	$C_{32}H_{22}N_6Na_2O_6S_2$	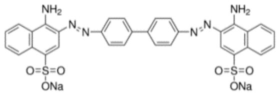	Anionic	696.66	499
Methylene blue	$C_{16}H_{18}N_3SCl\cdot 3H_2O$	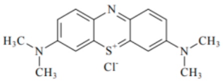	Cationic	373.90	664

Table S2. Repeatability test of Brunauer–Emmett–Teller (BET) surface area measurements.

AC Sample	AHL-AC6 (m^2/g)	IndL-AC (m^2/g)
Test 1	2018	1343
Test 2	2011	1391
Test 3		1378
**Mean**	**2015**	**1371**
**RSTD (%)**	**0.2**	**1.8**

Table S3. XPS measurements of surface elemental composition of lignin samples.

Samples	C (%)	O (%)	S (%)	P (%)	O/C
Indulin C	47.51	51.82	0.50	0.18	1.09
Alkali lignin	73.85	25.35	0.68	0.12	0.34
Commercial LS	60.16	37.01	2.69	0.14	0.62

Dye adsorption measurements and adsorption isotherm models.

Quantitative dye adsorptions were obtained by UV-Vis spectrophotometry through calibration. Congo red (CR) and methylene blue (MB) dye solutions of known concentrations were prepared. The absorbance at 499 and 664 nm for CR and MB, respectively, were measured. The calibration equation for CR (Equation S1) and MB (Equation (S2) were obtained as follow:

$$y = 0.05432x - 0.01812,\ r^2 = 0.9939 \quad \text{(S1)}$$

$$y = 0.17749x + 0.02691,\ r^2 = 0.9992 \quad \text{(S2)}$$

The percentage of dye removal (Y) and adsorption capacity (q_e) by an activated carbon (AC) sample were calculated from Equation (S3) and (S4):

$$Y(\%) = \left(1 - \frac{c_e}{c_0}\right) \times 100 \quad \text{(S3)}$$

$$q_e(\text{mg/g}) = \frac{c_0 - c_e}{m} v \quad \text{(S4)}$$

where c_0 and c_e are the initial and final concentration of dye (mg/L), respectively, v is the volume of dye solution (mL), and m is the mass of AC (g).

The equilibrium adsorption isotherms were fitted to the Langmuir model as expressed below [1].

$$q_e = \frac{q_m k_L c_e}{1+k_L c_e} \quad (S5)$$

$$\frac{c_e}{q_e} = \frac{1}{q_m k_L} + \frac{c_e}{q_m} \quad (S6)$$

where c_e is the concentration of adsorbate at equilibrium (mg/L), q_e is the equilibrium adsorption capacity (mg/g), q_m is the maximum adsorption capacity (mg/g) and k_L is the adsorption coefficient. The efficiency of adsorption can be predicted by the dimensionless equilibrium parameter R_L, which is defined as the Equation (S7) [2]:

$$R_L = \frac{1}{1+k_L c_0} \quad (S7)$$

where c_0 is the initial concentration of dyes (mg/L). The adsorption is considered as irreversible when $R_L = 0$, favorable when $0 < R_L < 1$, linear when $R_L = 1$, and unfavorable when $R_L > 1$.

The adsorption isotherms were also fitted to the Freundlich model expressed below by Equation (S8) and (S9) [3]:

$$q_e = k_F c_e^{1/n} \quad (S8)$$

$$\ln q_e = \ln k_F + \frac{1}{n}\ln c_e \quad (S9)$$

where k_F is the Freundlich constant as a measure of the degree of combination between the adsorbates and adsorbents and $1/n$ reflects the nonlinear degree of adsorption.

The adsorption kinetics that describes the solute uptake rate on the adsorbent and the possible mechanism of adsorption can be expressed using a pseudo-first order model based on solid capacity by Equation (S10) [4]:

$$\ln(q_e - q_t) = \ln q_e - k_1 t \quad (S10)$$

Or using a pseudo-second order model to predict the behavior over the whole range of adsorption by Equation (S11) [5]:

$$\frac{t}{q_t} = \frac{1}{k_2 q_e^2} + \frac{t}{q_e} \quad (S11)$$

where k_1 is the rate constant of the pseudo first-order adsorption (min^{-1}), k_2 is the rate constant of the pseudo second-order adsorption (g min/mg), t is the adsorption time (h), and q_t and q_e are the adsorption capacity at time t and at equilibrium, respectively (mg/g).

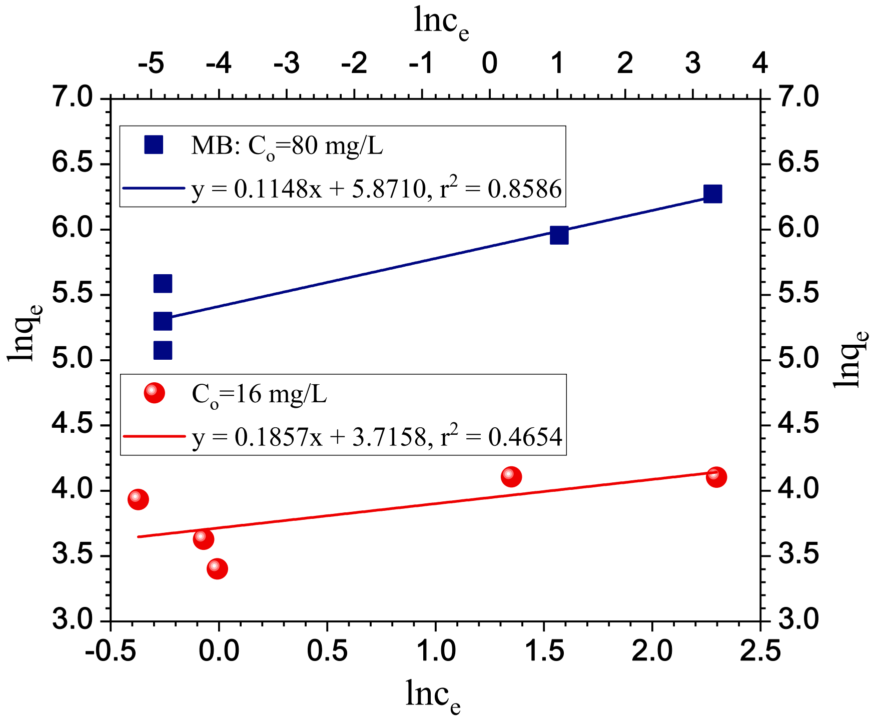

Figure S1. Adsorption isotherms of congo red (CR) and methylene blue (MB) by acid hydrotropic lignin (AHL)-activated carbon (AC)6 fit to the Freundlich model.

Table S4. Parameters of the Freundlich isotherm models for CR and MB adsorption by AHL-AC6.

Dye	1/n	k_F	R^2
CR	0.1857	41.1	0.4654
MB	0.1148	354.6	0.8586

Table S5. Comparisons of dye adsorption capacity by AC among different studies.

Adsorbents	S_{BET}	Adsorption capacity (mg/g)		T (°C) [c]	Sources
		$q_{m.cal}$[a]	$q_{m.exp}$[b]		
**CR**					
Bentonite/zeolite-NaP composite	512	46	44	-	6
ZnO nanorods loaded on AC	-	143	-	50	7
Guava leaves AC (H_3PO_4 activation)	-	48	40	30	8
Maghemite nanoparticles	82	208	-	25	9
Bael shell carbon (carbonized for 1 h at 450 °C)	-	98	-	30	10
Hollow Zn-Fe2O4 nanospheres	-	16	-	25	11
Carbon composite lignin-based adsorbent (glucose, calcium lignosulfonate and triethylene tetramine as raw materials)	-	299	-	25	12
Aminated alkali lignin (Mannich reaction using hexane-diamine and alkali lignin with the assistance of ultrasound)	-	71	-	25	13
Aminated calcium lignosulfonate (using triethylene tetramine)	12	258	-	25	14
Aluminum fumarate reduced graphene oxide (AlF-rGO)	952	133	179	25	15
Spent activated carbon (AC) loaded with zinc acetate (Microwave-assisted, CO_2 activation)	1100	17	-	35	16
Tomato stem AC ($FeCl_2$ activation)	971	159	-	25	17
AHL-AC6	2018	65	61	Room T	This study
**MB**					
Rice Straw Organosolv lignin (No further modifications)	-	40	39	20	18
Acetic acid lignin (Chemically Modified)	-	63	86	30	19
Activated lignin-chitosan composite extrudates	-	36	28	20	20
Commercial lignin (modified by 5-sulfosalicylic acid)	-	101	83	45	21

Grape processing wastes AC ($ZnCl_2$ activation)	1455	417	-	30	22
Prolifera AC ($ZnCl_2$ activation)	1688	270	263	25	23
P. oceanica (L.) dead leaves AC ($ZnCl_2$ activation)	1483	286	-	45	24
Bamboo-based AC (H_3PO_4 activation, heated with microwave)	1323	286	-	25	25
Date pits AC ($FeCl_3$ activation)	780	249	236	30	26
Sucrose AC (hydrothermal treatment to prepare spherical carbon, then KOH activation)	1534	704	709	25	27
Fruit shells AC	230	7.0		30	28
Kaolin material (industrially treated with sodium carbonate)	21	53	-	25	29
3D agar/graphene oxide composite aerogel	8	568	551	20	30
AHL-AC6	2018	535	530	Room T	This study

[a] Calculated maximum adsorption capacity by Langmuir model equation.
[b] Experimental maximum adsorption capacity.
[c] At which absorption was conducted.

Table S6. Parameters for the pseudo-first order and the pseudo-second order model equations of CR and MB adsorption.

Dyes	pseudo first-order model			pseudo second-order model		
	k_1	q_e	R^2	k_2	q_e	R^2
Congo Red (CR)	0.079	38.4	0.969	0.0059	48.9	0.971
Methylene Blue (MB)	0.224	68.8	0.914	0.0089	271.0	1.000

References:

1. Langmuir, I., The constitution and fundamental properties of solids and liquids. Part I. Solids. *Journal of the American chemical society* **1916,** 38, (11), 2221-2295.
2. Ngah, W. W.; Endud, C.; Mayanar, R., Removal of copper (II) ions from aqueous solution onto chitosan and cross-linked chitosan beads. *Reactive and Functional Polymers* **2002,** 50, (2), 181-190.
3. Freundlich, H., Over the adsorption in solution. *J. Phys. Chem* **1906,** 57, (385471), 1100-1107.
4. Weber, W. J.; Morris, J. C., Kinetics of adsorption on carbon from solution. *Journal of the sanitary engineering division* **1963,** 89, (2), 31-60.
5. Ho, Y.-S.; McKay, G., Pseudo-second order model for sorption processes. *Process biochemistry* **1999,** 34, (5), 451-465.
6. Shaban, M.; Abukhadra, M. R.; Shahien, M.; Ibrahim, S. S., Novel bentonite/zeolite-NaP composite efficiently removes methylene blue and Congo red dyes. *Environmental chemistry letters* **2018,** 16, (1), 275-280.
7. Ghaedi, M.; Biyareh, M. N.; Kokhdan, S. N.; Shamsaldini, S.; Sahraei, R.; Daneshfar, A.; Shahriyar, S., Comparison of the efficiency of palladium and silver nanoparticles loaded on activated carbon and zinc oxide nanorods loaded on activated carbon as new adsorbents for removal of Congo red from aqueous solution: Kinetic and isotherm study. *Materials Science Engineering: C* **2012,** 32, (4), 725-734.
8. Ojedokun, A. T.; Bello, O. S., Kinetic modeling of liquid-phase adsorption of Congo red dye using guava leaf-based activated carbon. *Applied Water Science* **2017,** 7, (4), 1965-1977.

9. Afkhami, A.; Moosavi, R., Adsorptive removal of Congo red, a carcinogenic textile dye, from aqueous solutions by maghemite nanoparticles. *Journal of Hazardous Materials* **2010,** 174, (1-3), 398-403.
10. Ahmad, R.; Kumar, R., Adsorptive removal of congo red dye from aqueous solution using bael shell carbon. *Applied Surface Science* **2010,** 257, (5), 1628-1633.
11. Rahimi, R.; Kerdari, H.; Rabbani, M.; Shafiee, M., Synthesis, characterization and adsorbing properties of hollow Zn-Fe2O4 nanospheres on removal of Congo red from aqueous solution. *Desalination* **2011,** 280, (1-3), 412-418.
12. Wang, X.; Jiang, C.; Hou, B.; Wang, Y.; Hao, C.; Wu, J., Carbon composite lignin-based adsorbents for the adsorption of dyes. *Chemosphere* **2018,** 206, 587-596.
13. Wang, X.; Zhang, Y.; Hao, C.; Dai, X.; Zhou, Z.; Si, N., Ultrasonic-assisted synthesis of aminated lignin by a Mannich reaction and its decolorizing properties for anionic azo-dyes. *RSC advances* **2014,** 4, (53), 28156-28164.
14. Wang, Y.; Zhu, L.; Wang, X.; Zheng, W.; Hao, C.; Jiang, C.; Wu, J., Synthesis of aminated calcium lignosulfonate and its adsorption properties for azo dyes. *Journal of industrial and engineering chemistry* **2018,** 61, 321-330.
15. Azhdari, R.; Mousavi, S. M.; Hashemi, S. A.; Bahrani, S.; Ramakrishna, S., Decorated graphene with aluminum fumarate metal organic framework as a superior non-toxic agent for efficient removal of Congo Red dye from wastewater. *Journal of Environmental Chemical Engineering* **2019,** 7, (6), 103437.
16. Qu, W.; Hu, Q.; Zhu, Y.; Peng, J.; Zhang, L., Microwave-assisted regeneration of spent activated carbon containing zinc acetate and its application for removal of congo red. *Desalination and Water Treatment* **2016,** 57, (58), 28496-28511.
17. Fu, K.; Yue, Q.; Gao, B.; Wang, Y.; Li, Q., Activated carbon from tomato stem by chemical activation with FeCl2. *Colloids and Surfaces A: Physicochemical and Engineering Aspects* **2017,** 529, 842-849.
18. Zhang, S.; Wang, Z.; Zhang, Y.; Pan, H.; Tao, L., Adsorption of methylene blue on organosolv lignin from rice straw. *Procedia Environmental Sciences* **2016,** 31, 3-11.
19. Feng, Q.; Cheng, H.; Chen, F.; Zhou, X.; Wang, P.; Xie, Y., Investigation of cationic dye adsorption from water onto acetic acid lignin. *Journal of Wood Chemistry and Technology* **2016,** 36, (3), 173-181.
20. Albadarin, A. B.; Collins, M. N.; Naushad, M.; Shirazian, S.; Walker, G.; Mangwandi, C., Activated lignin-chitosan extruded blends for efficient adsorption of methylene blue. *Chemical Engineering Journal* **2017,** 307, 264-272.
21. Jin, Y.; Zeng, C.; Lü, Q.-F.; Yu, Y., Efficient adsorption of methylene blue and lead ions in aqueous solutions by 5-sulfosalicylic acid modified lignin. *International journal of biological macromolecules* **2019,** 123, 50-58.
22. Sayğılı, H.; Güzel, F.; Önal, Y., Conversion of grape industrial processing waste to activated carbon sorbent and its performance in cationic and anionic dyes adsorption. *Journal of Cleaner Production* **2015,** 93, 84-93.
23. Li, Y.; Du, Q.; Liu, T.; Peng, X.; Wang, J.; Sun, J.; Wang, Y.; Wu, S.; Wang, Z.; Xia, Y., Comparative study of methylene blue dye adsorption onto activated carbon, graphene oxide, and carbon nanotubes. *Chemical Engineering Research and Design* **2013,** 91, (2), 361-368.
24. Dural, M. U.; Cavas, L.; Papageorgiou, S. K.; Katsaros, F. K., Methylene blue adsorption on activated carbon prepared from Posidonia oceanica (L.) dead leaves: Kinetics and equilibrium studies. *Chemical Engineering Journal* **2011,** 168, (1), 77-85.
25. Liu, Q.-S.; Zheng, T.; Li, N.; Wang, P.; Abulikemu, G., Modification of bamboo-based activated carbon using microwave radiation and its effects on the adsorption of methylene blue. *Applied Surface Science* **2010,** 256, (10), 3309-3315.
26. Theydan, S. K.; Ahmed, M. J., Adsorption of methylene blue onto biomass-based activated carbon by FeCl3 activation: Equilibrium, kinetics, and thermodynamic studies. *Journal of Analytical and Applied Pyrolysis* **2012,** 97, 116-122.
27. Bedin, K. C.; Martins, A. C.; Cazetta, A. L.; Pezoti, O.; Almeida, V. C., KOH-activated carbon prepared from sucrose spherical carbon: Adsorption equilibrium, kinetic and thermodynamic studies for Methylene Blue removal. *Chemical Engineering Journal* **2016,** 286, 476-484.
28. Aboua, K. N.; Yobouet, Y. A.; Yao, K. B.; Gone, D. L.; Trokourey, A., Investigation of dye

adsorption onto activated carbon from the shells of Macoré fruit. *Journal of environmental management* **2015,** 156, 10-14.
29. Mouni, L.; Belkhiri, L.; Bollinger, J.-C.; Bouzaza, A.; Assadi, A.; Tirri, A.; Dahmoune, F.; Madani, K.; Remini, H., Removal of Methylene Blue from aqueous solutions by adsorption on Kaolin: Kinetic and equilibrium studies. *Applied Clay Science* **2018,** 153, 38-45.
30. Chen, L.; Li, Y.; Du, Q.; Wang, Z.; Xia, Y.; Yedinak, E.; Lou, J.; Ci, L., High performance agar/graphene oxide composite aerogel for methylene blue removal. *Carbohydrate polymers* **2017,** 155, 345-353.
